# Pediatric Heart Failure, Lagging, and Sagging of Care in Low Income Settings: A Hospital Based Review of Cases in Ethiopia

**DOI:** 10.1155/2016/7147234

**Published:** 2016-11-16

**Authors:** Solmon Gebremariam, Tamirat Moges

**Affiliations:** ^1^Department of Pediatrics and child Health, Mekelle University, Mek'ele, Ethiopia; ^2^Department of Pediatrics and Child Health, School of Medicine, Addis Ababa University, Addis Ababa, Ethiopia

## Abstract

*Introduction*. Causes of acute heart failure in children range from simple myocarditis complicating chest infection to complex structural heart diseases.* Objective.* To describe patterns, predictors of mortality, and management outcomes of acute heart failure in children.* Methods.* In retrospective review, between February 2012 and October 2015 at a tertiary center, 106 admitted cases were selected consecutively from discharge records. Data were extracted from patients chart and analyzed using SPSS software package. *t*-test and statistical significance at *P* value < 0.05 with 95% CI were used.* Result*. Acute heart failure accounted for 2.9% of the total pediatric admissions. The age ranged from 2 months up to 14 years with mean age of 8 years. Male to female ratio is 1 : 2.1. Rheumatic heart disease accounted for 53.7%; pneumonia, anemia, infective endocarditis, and recurrence of acute rheumatic fever were the main precipitating causes. Death occurred in 19% of cases. Younger age at presentation, low hemoglobin concentration, and undernutrition were associated with death with *P* value of 0.00, 0.01, and 0.02, respectively.* Conclusions and Recommendation*. Pediatric heart failure in our settings is diagnosed mainly in older age groups and mostly precipitated due to preventable causes. Significant mortality is observed in relation to factors that can be preventable in children with underlying structural heart disease. Early suspicion and diagnosis of cases may reduce the observed high mortality.

## 1. Introduction

Heart failure is a clinical entity where the heart does not function to its level best as it does in its healthy state [1]. Heart failure is a common cause of morbidity and mortality in pediatric patients in the third world [2]. Change in the prevalence, pattern, and etiologies among the different ages, geographic area, and social classes creates difficulties to come up with formidable research in pediatrics. Compared with adult patients, where heart failure resulted from an insult to the myocardium, heart failure in children occurs due to heart lesions that cause volume overload as in large ventricular septal defect or due to lesions that causes obstruction to flow as in aortic stenosis [3]. Often it is hard to diagnose heart failure in children as the clinical manifestations have significantly overlap with other pathologic conditions. Pediatric heart failure has significant consequences: it is associated with increased mortality, prolonged hospital stay, and increased economic and social burden to the family [2]. Acute heart failure in children in the developed communities is often due to cardiomyopathic causes or palliated congenital heart disease as opposed to developing countries where unoperated congenital heart disease and acquired heart disease are more prevalent [4]. There is also great need for evidence based management guidelines in pediatric heart failure, because the current guidelines in pediatric heart failure management are mainly derived from adult studies [5]. Clinical profiles of heart failure are not well described in the sub-Saharan Africa, though few countries reported childhood heart failure [6, 7]. In Ethiopia although few reports on the patterns of heart disease in children are available, studies on topic of childhood heart failure are nonexistent to our knowledge [8, 9]. Therefore, we sought to describe acute heart failure in children at a tertiary hospital in Addis Ababa, Ethiopia.

*Objective*. The objective of this paper is to describe patterns, association for mortality, and management outcomes of acute heart failure in children. Method: it includes study design which was a hospital based retrospective review of admitted cases and setting which is Tikur Anbessa Specialized Hospital (TASH), a tertiary referral center in Ethiopia in the pediatric medicine. The hospital is located in the center of Addis Ababa the capital city; Department of Pediatrics has eight different service units to which these patient groups can be referred. The hospital has an average of 170 pediatric beds where 25–30 beds are occupied by pediatric cardiac admissions. Patients were coming from all corners of Ethiopia as the hospital is the biggest tertiary referral center.

*Sample*. Patients' chart review was performed on 106 consecutive discharged patients with acute heart failure from February 2012 to October 2015. Patients under the age of 15 years from pediatric wards and emergency care unit with the discharge diagnosis of acute heart failure were included. 

*Inclusion Criteria*. The clinical criteria for heart failure in a pediatric patient were made if any three of the following were fulfilled: (1) significant tachycardia for age (heart rate > 160 bpm in infancy, >140 bpm at the age of 2 years, >120 bpm at the age of 4 years, and >100 bpm above 6 years of age); (2) significant tachypnoea for age (respiratory rate > 60/min in newborn, >40/min in <24 months of age, >30/min in 2–5 years of age, >28/min in 5–10 years of age, and >25/min >10 years of age); (3) cardiomegaly (displaced apical beat with a central trachea or cardiothoracic ratio >60% in <1 year of age, >55% in 1–5 years of age, and >50% in >5 years of age); (4) tender hepatomegaly of at least 3 cm size below the right costal margin [10].

Independent variables included age, sex, duration of symptoms, underlying heart disease, precipitating cause, left ventricular systolic function (LV EF), right ventricular systolic function, date of start of treatment, type of treatment, and noncardiac treatment. Outcome variables included total pediatric admissions during study period, prevalence and etiology of heart failure, severity of heart failure, growth failure, pulmonary hypertension, recurrent chest infections (pneumonia), length of hospital stay, number of patients with improvement on discharge, mortality rate, and being defaulted.

Cases with incomplete information and age above 14 years were excluded from the study. Sample size was determined based on the prevalence of heart failure in sub-Saharan African countries using a single population sample size calculation method. Questionnaires were developed based on desired information. Each query was appropriately pretested to determine appropriate information. Based on available data, information about the onset of illness, the presenting complaint, duration of illness, treatments received, and other pertinent clinical features were obtained. The type and severity of cardiac lesions were ascertained on the basis of physical examination and radiologic and electrocardiographic data, including echocardiography. The cardiac unit is equipped with 4 modern echocardiographic machines with pediatric probes, ECG machines, pulse oximeter devices.

All cases were classified according to etiology, age, and clinical characteristics. Congenital and rheumatic heart diseases were defined according to the specific criteria available for their classifications [11, 12]. Diagnosis of congenital as well as acquired heart disease is confirmed by echocardiographic examination (2D, M-mode, and color-doppler mode imaging).

Data were extracted from the patient charts and were filled into the questionnaire format by the investigators. Data quality was checked for its completeness, corrected on the spot for information that appeared inconsistent. To ensure consistency, double data entry was used. Data concerning number of pediatric visits, pediatric admissions, and cardiac cases during the study period were obtained from the record office.

Data were entered into SPSS version 20, IBM, USA, and Excel 2013. Categorical data were presented as percentages or proportions. A chi-square test is used to determine whether an association between two categorical variables is significant or not. Continuous variables were analyzed using mean, median, and standard deviations. Student's *t*-test was used to compare mean values. Statistical significance was considered when *P* value falls below 0.05 at 95% confidence interval. Results were presented in tables and figures.

*Operational Definition*. Congenital heart disease (CHD) is a problem with the heart structure and function that is present at birth. Rheumatic valvular heart disease (RVHD) is active or inactive disease of the heart that results from rheumatic fever and is characterized by reduced functional capacity caused by inflammatory changes in the myocardium or scarring of the valves. Mild anemia is defined as a hemoglobin concentration in the range of 9.5–11 gm/dL. Moderate anemia is defined as hemoglobin in the range of 8–9.5 gm/dL. Severe anemia is defined as hemoglobin concentration < 8 gm/dL [13]. Treatment is defined in this particular case as medical treatments given excluding surgery and/or catheter intervention of the cardiac lesions. Left ventricular systolic dysfunction is considered when the left ventricular ejection fraction falls below 55% as determined by 2D-echocardiography [14, 15]. Pneumonia in a child with congestive heart failure is diagnosed in the presence of fever, increased in chest X-ray evidence of lung parenchymal infiltrates and elevated white blood cell count in the presence of evidence for acute heart failure. Severe acute heart failure for children 0–5 years of age is considered if symptoms occurred at rest with tachypnea, retractions, grunting, or diaphoresis. For children above the age of 5 years, severe acute heart failure is referred to as NYHA classes III and IV [10, 16]. Management outcome refers to the discharge outcome. Malnutrition is classified based on weight for height percentile of the median: (1) adequate weight for height measurement is defined when the percentile falls between 90 and 120%; (2) mild wasting is considered when the percentile value falls between 80 and 89% of the median value; (3) moderate wasting is considered when the percentile value falls between 70 and 79% of the median value; (4) severe wasting is considered when the percentile value < 70% of the median value [17, 18]. The study was approved by the department research and promotion committee.

## 2. Result

During the study period, 3672 pediatric admissions were registered to the different pediatric units. Out of these, there were 106 cases of acute heart failure. The prevalence of acute heart failure was 2.9% of all pediatric admissions. The mean age at presentation was 8 years. All cases were classified in New York Heart Association (NYHA) or Rose functional class IV severity grade.  Table 1 shows the distribution of different age categories. Over 65% of the cases were above the age of 6 years. Children below the age of 3 years accounted only for 20% of the cases. Female sex predominates in all age categories. From the same table, one can see that rheumatic heart disease accounted for over half of the cases. Mitral and aortic valves were involved in 100% and 31% of the cases, respectively. Congenital plus acquired nonrheumatic heart diseases cases accounted for the remainder of the cases. Systolic dysfunction was observed in 8.5% and 24.5% of the right and the left ventricular muscles, respectively. Earlier age at presentation, low body weight (kg), and low hemoglobin concentration were shown to be predictors of mortality as seen in Table 2.

**Figure 1:**
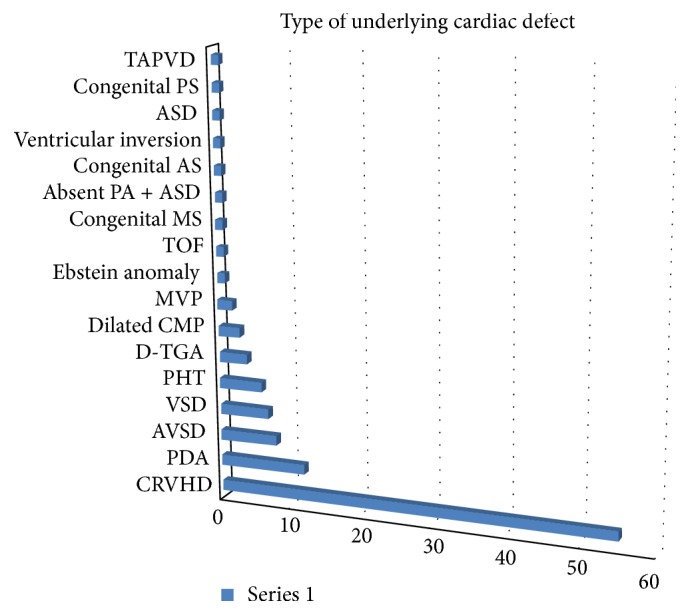
Frequency of underlying cardiac lesions; childhood heart failure at Tikur Anbessa Specialized Hospital, 2016. ASD: atrial septal defect, As: aortic stenosis, PA: pulmonary artery, MS: mitral stenosis, TOF: tetralogy of Fallot, MVP: mitral valve prolapse, CMP: cardiomyopathy, D-TGA: D-type transposition of the great arteries, PHT: pulmonary hypertension, VSD: ventricular septal defect, AVSD: atrioventricular septal defect, PDA: patent ductus arteriosus, CRVHD: chronic valvular heart disease, TAPVD: total anomalous pulmonary venous drainage, and PS: pulmonary stenosis.

**Figure 2:**
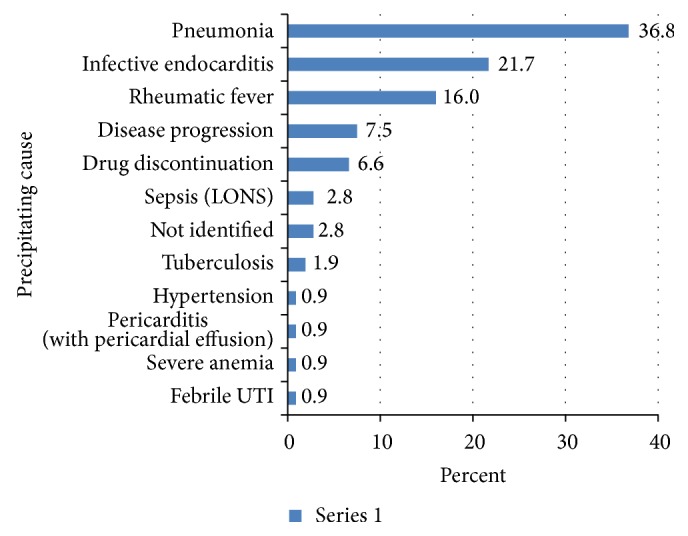
Precipitating causes of childhood heart failure at Tikur Anbessa Specialized Hospital, 2016. LONS: late onset neonatal sepsis and UTI: urinary tract infection.

**Figure 3:**
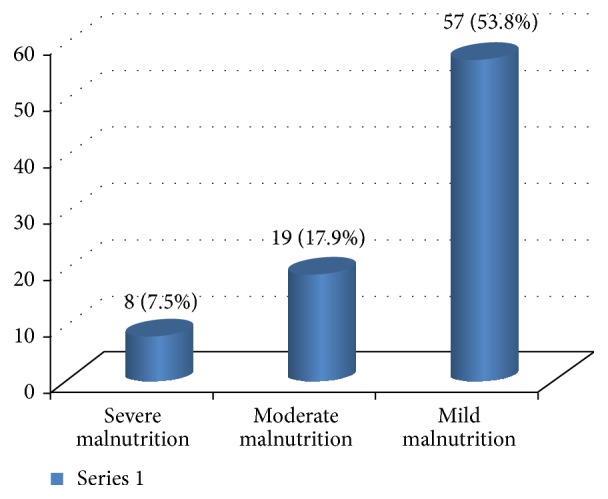
Weight for height status of cases of childhood heart failure at Tikur Anbessa Specialized Hospital, 2016. *y*-axis: percentage of patients.

Figure 1 shows frequency of underlying cardiac conditions that predisposes to acute heart failure. Heart failure was precipitated by the list of factors displayed in Figure 2. Pneumonia, infective endocarditis, and rheumatic fever recurrence were the most common causes of heart failure in decreasing order. Weight for height status is also shown in Figure 3 with mild, moderate, and severe wasting observed in 53.8%, 17.9%, and 7.5% of cases, respectively.

**Figure 4:**
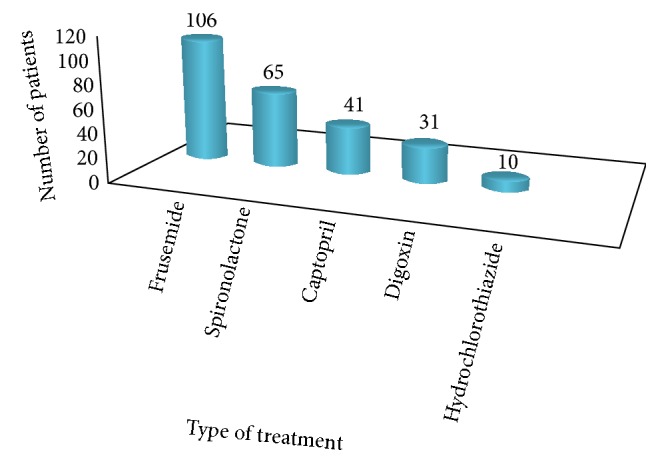
Drug treatment profile of cases in TASH 2016. *y*-axis: number of patients.

Medication profile of cases is displayed in Figure 4. Furosemide was prescribed for all cases at the time of admission, whereas spironolactone, captopril, digoxin, and hydrochlorothiazide were prescribed in 65%, 41%, 31%, and 10%, respectively. Beta blockers were not part of the medication lists. Seventy-five percent of the cases were discharged improved, 19% died, and the rest of them were either defaulted or returned back to their original institution to complete their medications.

## 3. Discussion

This hospital based retrospective chart review represented the general population of patients that sought care at Tikur Anbessa Hospital from all regions of Ethiopia. Data collection was reliable as the investigators coded all the diagnoses. The mean age at first presentation was low for mortality cases. This may have to do with the severity of underlying cardiac disease. Some of the findings were unique in our setting compared to reports from international sources. For example, infants and young children accounted for low proportion of the cases in this review. Massin et al. reported in one review that 58.1% of their study subjects were infants [19] In another study, 75.7% of deaths due to acute heart failure occurred in patients younger than 1 year of age [4, 20]. In a report of pediatric acute heart failure from Kenya, the reported mean age was 4.7 years, with a peak at 1–3 years. In another report of childhood heart failure from Calabar, Nigeria, 69% percent of the patients were aged between 1 and 5 years [6]. One possible explanation for this observation in our settings may be the challenge of diagnosing heart failure in small children. Other clinical conditions may have a similar clinical presentation, for example, respiratory tract infections. In many occasions, an infant may present with both heart failure and pneumonia. Thus, there is a tendency among the staff to record pneumonia as final diagnosis and omit heart failure because health providers are not confident enough in the diagnosis of acute heart failure in small children. Hyperactive air way disease can also be confused with heart failure in infants. The study subjects in the current study were the unoperated cases of cardiac structural problems. Therefore, those patients with severe structural cardiac disease may have died early, with only those with less severe structural disease presenting later in life. This is suggested in Table 2.

In this review, female sex appears to be more commonly reported at all age groups. It appears from many studies that female sex has higher risk of developing cardiovascular disease compared to male sex [21]. It has been reported that sex disparities in cardiovascular health exist among women of all ages, socioeconomic backgrounds, and racial subgroups. According to Singh, parental preference for male children exists in many societies, where girls with heart disease are not provided with the same treatment opportunities as boys. Based on suggestion of Navin and Nanda, there may also be an underlying biological sex difference [22] However, Julius A Ogeng'o et al. from Kenya reported male preponderance in their report of 158 cases [23]. Other studies suggest that there is increased female preponderance for mitral stenosis in patient with known rheumatic heart disease [22, 24]. All these factors may explain our findings of female predominance. Yet more evidence is required to explain these observations.

One of the prevailing findings in the current review is coexisting infections. Several studies reported respiratory infections to be common with acute heart failure particularly in children with structural heart disease [7, 25]. The mechanism by which recurrent chest infections cause heart failure has been postulated by Sadoh. It was suggested that, with pulmonary overcirculation and pulmonary edema associated with large left to right shunting of blood, pulmonary congestion ultimately becomes a focus of infection in the lower respiratory tract [26].

Children with rheumatic heart disease in Ethiopia are at risk of developing recurrence of rheumatic fever, as many of them are not receiving appropriate secondary prophylaxis with penicillin [8]. It is reported that the risk of rheumatic fever recurrence is significantly high if a patient is taking less than 80% of the prescribed doses [27]. On the other hand, dental health and dental hygiene are often compromised among communities. It is also known that children with congenital and/or acquired valvular heart lesions are at high risk of developing infective endocarditis, especially if they are not covered with subacute bacterial endocarditis prophylaxis during certain invasive procedures. This problem is well reflected in our previous report [9]. Over half the cases in our current study had laboratory evidence of anemia. A similar observation was made in adult patients with heart failure by Tang et al. They reported that up to one-fifth to one-third of heart failure cases had developed anemia. Accordingly, the prevalence of anemia increases with severity of the heart failure [28]. On the other hand, Worens Luiz reported the prevalence of anemia in the study population to be 41.0% [29]; Lindenfeld reported that anemia is consistently associated with poorer survival in all patient populations [30]. The high prevalence of anemia in our current study may be related to NYHA or Ross class IV severity grading. Plasma volume expansion that occurs in congestive heart failure may also contribute to anemia by a process of hemodilution. Chronic kidney disease is common comorbidity in heart failure and is strong independent predictor of anemia [31]. According to Tang, any abnormality that reduces renal secretion or bone marrow responses to erythropoietin may result in anemia. We were unable to collect this information in our current report. We would like to emphasize the importance of kidney evaluation in all patients admitted with heart failure. It was also reported that patients with cardiac cachexia are at increased risk for anemia. Reports suggest that serum level of proinflammatory cytokines is increased in cachexic patients with congestive heart failure and may contribute to development of anemia by several mechanisms [32]. Significant weight differences correlated with increased mortality in the current study. Seventy-nine percent of the patients had malnutrition based on weight for height measurements. According to a report of heart failure patients in adults by Amare et al. from Jimma University, in South-West Ethiopia, 77.8% of the these patients were malnourished [33]. It has been reported that the risk of death associated with heart failure is significantly increased in patients at risk of malnutrition or with concomitant malnutrition [34]. Coatsa followed the one-year mortality rate of patients in a prospective study, who were classified as malnourished according to the “Mini Nutritional Assessment score.” A one-year mortality rate was found to be 56% in malnourished patients, compared to 23.5% in patients at risk of malnutrition and 11.3% in those patients with an adequate nutritional status [35].

Malnutrition in heart failure has been ascribed to neurohormonal alterations, especially anabolic/catabolic imbalance and increased cytokine release. Anorexia becomes worse, particularly during acute decompensation of heart failure [32]. Hormonal factors related to heart failure can affect appetite, further interfering with nutrition. According to the discussion by Niya Jones, chronic heart failure interferes with the absorption of fats and protein in particular; diminished blood flow to the intestines and fluid accumulation, called gut edema, contribute to malabsorption and likely promote the development of cardiac cachexia [36]. In general, children with acute heart failure have increased work of breathing and increased cardiac activity to compensate for the low cardiac function. This exposes them to high energy expenditure in the face of limited intake leading to cardiac cachexia.

The number of cases who died in the current study is relatively higher compared to most reports in the sub-Saharan African countries and most others outside the region. We already discussed the role of malnutrition and anemia in increasing risk of death in heart failure. However, studies from other sub-Saharan African countries did not report the role of malnutrition in their reviews. In adult studies, the rate of malnutrition was not as high as seen in our current report. The high prevalence of undernutrition, anemia and coinfection in our context may explain the high mortality rate observed [6, 23].

We observed in our study that captopril treatment was initiated with the lowest starting dose of 0.1 to 0.3 mg/kg/dose once (OD) daily up to three times daily (TID); however, escalation of dose was not subsequently done. Therefore, angiotensin converting enzyme inhibitor (ACE-inhibitor) therapy was, in general, suboptimal in our setting. However, literature recommends that, following a starting dose, a tittered dose as high as 0.5−2 mg/kg every 8 hours can be used in pediatric heart failure [37, 38]. The observed treatment disparity in comparison to the standard practice is, we believe, a call to prepare management protocols for our interns and residents. Therapy with beta blockers was not used in any of our patients. Our result differs from the Kenyan and Nigerian study in the mean age and gender of cases described. Surprisingly our result is similar to most reports in adult patients. This may be due to the fact that we have described older patients in contrast to the Kenyan or the Nigerian patients groups [6, 7].

## 4. Conclusion

Age distribution of pediatric heart failure in our setting is dominated by older age population. Acute heart failure in infants and small children may have been underreported, due to misdiagnosis or early death of patients. Female sex predominance observed in adult reports is also noted in the current study. Underlying cardiac defects predisposing patients to heart failure were dominated by rheumatic heart disease in this review. This highlights the importance of national mobilization against this preventable condition. Undernutrition, anemia and infections were also important preventable comorbid conditions predisposing to death of cases.

## Figures and Tables

**Table 1:** Patient characteristics of childhood heart failure at Tikur Anbessa Specialized Hospital, 2016.

	Male	Female	Total
Age			
0–36 months	5 (14.7%)	16 (22.2%)	21 (19.8%)
37–72 months	4 (11.8%)	12 (16.7%)	16 (15.1%)
73–108 months	4 (11.8%)	16 (16.7%)	20 (19.0%)
109–168 months	21 (61.8%)	28 (38.9%)	49 (46.0%)
Type of heart disease			
CHD	12 (41.7%)	30 (35.3%)	42 (39.6%)
RVHD	19 (52.8%)	38 (55.9%)	57 (53.7%)
Other causes	3 (5.6%)	4 (8.8%)	7 (6.6%)
RVSD	3 (2.8%)	6 (5.7%)	9 (8.5%)
LVSD	8 (7.5%)	18 (17%)	26 (24.5%)

CHD: congenital heart disease, RVHD: rheumatic valvular heart disease, RVSD: right ventricular systolic dysfunction, and LVSD: left ventricular systolic dysfunction.

**Table 2:** Predictors of mortality based on outcome; childhood heart failure at Tikur Anbessa Specialized Hospital, Addis Ababa, Ethiopia, 2016.

variable	Outcome		*P* value
	Death	Discharged	
Mean age at presentation in years	5.9	9.4	0.001
Mean duration of symptoms (days)	15.2	19.3	0.5
Mean duration of hospital stay (days)	16.7	24.8	0.05
Mean weight (Kg)	16.4	20.5	0.02
LV ejection fraction% (mean)	39.0	36.8	0.64
Mean hemoglobin level (gm/dl)	8.7	10.3	0.01
Mean furosemide dose in mg/kg	1.1	1.0	0.97
Mean digoxin dose/kg	0.006	0.007	0.36
Mean spironolactone dose/kg	0.9	0.9	0.70
Mean dose of captopril/kg	0.4	0.4	0.35

## Additional Points

*Recommendation*. Presence of undernutrition, anemia and infections should be evaluated in all cardiac patients and should be treated early. Delayed suspicion and detection of congenital heart disease may be prevented by careful auscultation of the heart during medical evaluation of every child by a primary care physician. Primary and secondary prophylaxis of acute rheumatic fever should be a national agenda.
